# Phage Therapy Potentiates Second-Line Antibiotic Treatment against Pneumonic Plague

**DOI:** 10.3390/v14040688

**Published:** 2022-03-26

**Authors:** Yaron Vagima, David Gur, Moshe Aftalion, Sarit Moses, Yinon Levy, Arik Makovitzki, Tzvi Holtzman, Ziv Oren, Yaniv Segula, Ella Fatelevich, Avital Tidhar, Ayelet Zauberman, Shahar Rotem, Emanuelle Mamroud, Ida Steinberger-Levy

**Affiliations:** 1 Department of Biochemistry and Molecular Genetics, Israel Institute for Biological Research, Ness-Ziona 74100, Israel; yaronv@iibr.gov.il (Y.V.); gurd@iibr.gov.il (D.G.); moshea@iibr.gov.il (M.A.); sarit5761@gmail.com (S.M.); yinonl@iibr.gov.il (Y.L.); zivo@iibr.gov.il (Z.O.); avitalt@iibr.gov.il (A.T.); ayeletz@iibr.gov.il (A.Z.); shaharr@iibr.gov.il (S.R.); 2 Department of Biotechnology, Israel Institute for Biological Research, Ness-Ziona 74100, Israel; arikm@iibr.gov.il (A.M.); tzvih@iibr.gov.il (T.H.); yanivse@iibr.gov.il (Y.S.); ellaf@iibr.gov.il (E.F.)

**Keywords:** phage therapy, antibiotic therapy, ceftriaxone, *Yersinia pestis*, plague, antibiotic resistance, фA1122, PST, phage–antibiotic combination

## Abstract

Plague pandemics and outbreaks have killed millions of people during the history of humankind. The disease, caused by the bacteria *Yersinia pestis*, is currently treated effectively with antibiotics. However, in the case of multidrug-resistant (MDR) bacteria, alternative treatments are required. Bacteriophage (phage) therapy has shown efficient antibacterial activity in various experimental animal models and in human patients infected with different MDR pathogens. Here, we evaluated the efficiency of фA1122 and PST phage therapy, alone or in combination with second-line antibiotics, using a well-established mouse model of pneumonic plague. Phage treatment significantly delayed mortality and limited bacterial proliferation in the lungs. However, the treatment did not prevent bacteremia, suggesting that phage efficiency may decrease in the circulation. Indeed, *in vitro* phage proliferation assays indicated that blood exerts inhibitory effects on lytic activity, which may be the major cause of treatment inefficiency. Combining phage therapy and second-line ceftriaxone treatment, which are individually insufficient, provided protection that led to the survival of all infected animals—a synergistic protective effect that represents a proof of concept for efficient combinatorial therapy in an emergency event of a plague outbreak involving MDR *Y. pestis* strains.

## 1. Introduction

*Yersinia pestis*, a Gram-negative bacterium and the etiological agent of plague, is a lethal pathogen that has led to three global pandemics throughout human history [1,2]. Although natural plague outbreaks are rare, plague is considered a reemerging disease [3,4]. A recent large outbreak occurred in Madagascar in 2017, where many patients contracted the pneumonic form of the disease [5,6]. Due to its lethality and the potential for person-to-person infectivity, *Y. pestis* is classified by the Centers for Disease Control and Prevention (CDC) as a Tier 1 select agent [1,7]. Natural exposure of humans to the bacteria may occur through a carrier flea bite, leading to bubonic or septic plague that might further develop into a secondary pneumonic plague, or by air transmission, leading to primary pneumonic plague [1]. Pneumonic plague is a rapidly deteriorating disease that leads to death if the patient is not immediately treated with the recommended antibiotic [1,3].

*Y. pestis* strains are usually susceptible to the recommended antibiotics, including streptomycin, gentamicin, levofloxacin, ciprofloxacin and doxycycline [8]. However, several strains isolated from rodents or from patients with plague exhibit resistance to first-line antibiotics, such as streptomycin [3,9,10,11,12]. Moreover, a major concern exists regarding the possibility of the generation and usage of antibiotic-resistant *Y. pestis* strains in a bioterror event [3,4,7]. Currently, no licensed plague vaccine is available in Western countries [4,13], thus emphasizing the importance of developing alternative treatments, such as bacteriophage (phage) therapy.

Lytic phages are bacterial viruses with high specificity toward their hosts. Phage replication inside the host bacteria leads to bacterial lysis and cell death, releasing progeny virions in the so-called “lytic cycle”. The potential of using phages for antimicrobial therapy has been known for more than a century, since phages were independently identified by Frederik Twort (in 1915) and Felix d’Herelle (in 1917) [14]. Their advantages of selective and efficient bacterial killing, self-replication in the host, safety as treatments and simple and inexpensive preparation led to their application in the 1920s as a treatment for various infectious diseases, such as dysentery and cholera [14,15]. However, the appearance of commercialized antibiotics in the late 1940s changed the paradigm, and Western countries preferentially used antibiotics, which are characterized chemical molecules, rather than phages, which are biological viral entities [14]. In contrast to the situation in the West, phage therapy continued to serve as an acceptable remedy in the former USSR, Georgia and Eastern European countries [16]. Recently, as antibiotic resistance has become a global threat to public health, renewed interest in phage therapy has emerged in Western countries. Phage efficiency and safety have been investigated using various bacterial-infected animal models and by conducting human clinical trials [17]. Moreover, an increasing number of case reports have described successful compassionate-use treatment of multidrug-resistant (MDR) bacterial-infected patients by combining antibiotics with lytic phages (see [18,19] and recent reviews [17,20]).

In the present study, we evaluated the potential efficacy of phage therapy for pneumonic plague using a well-established mouse model. The therapeutic potential of treatment with the *Y. pestis*-specific lytic phages фA1122 and PST was assessed alone, as a dual cocktail, or in combination with the second-line broad-spectrum cephalosporin antibiotic ceftriaxone. Both phages were chosen for treatment, as they bind to the lipopolysaccharide (LPS) molecule, and mutations in this molecule attenuate *Y. pestis* virulence [21,22], thus reducing the possibility of the development of phage resistance.

## 2. Materials and Methods

### 2.1. Bacterial Strains, Bacteriophages and Growth Media

*Y. pestis* strains used in this study are listed in Table 1. The Kim53 and Kim53Δ70Δ10 strains were grown on Brain Heart Infusion Agar (BHIA; BD, MD, USA), and Kim53-*lux* and EV76-*lux* were routinely grown on BHIA supplemented with ampicillin (200 µg/mL, A0166, Sigma–Aldrich, Rehovot, Israel).

The *Y. pestis*-specific lytic phages used in this study were фA1122 (accession no. NC004777, kindly provided by professor Mikael Skurnik [26]), and PST (ATCC, cat. no.: 23207-B1; accession no. KF208315).

### 2.2. Animal Studies

The study was performed in accordance with the Recommendations for the Care and Use of Laboratory Animals (National Institutes of Health (NIH)). Animal experiments were performed in accordance with Israeli law and were approved by the Institutional Ethics Committee for animal experiments (protocol nos.: M-68-17, M-59-18, M-47-19, M-01-20, M-72-20 and M-25-21). Experiments were performed in an animal biosafety level 3 (ABSL-3) laboratory. Eight- to 12-week-old female mice (C57BL/6J; Invigo, Israel) were used in all animal experiments.

For intranasal (IN) challenge, bacterial colonies of Kim53 or Kim53-*luxCDABE* were grown on heart infusion broth (HIB, BD, Cockeysville, MD, USA) supplemented with 0.2% xylose and 2.5 mM CaCl_2_ (Sigma–Aldrich, Rehovot, Israel) at 28 °C for 22 h, as previously described [24]. Counting of colony-forming units (CFUs) was performed by plating 0.1 mL of the appropriate culture dilutions on BHIA plates and incubating them for 48 h at 28 °C. Prior to infection, mice were anesthetized with a mixture of 0.5% ketamine HCl and 0.1% xylazine and then infected IN with 35 μL of the bacterial suspension containing 10 × LD_50_ or 100 × LD_50_ Kim53 (1xINLD_50_ = 1100 CFU [27]) per mouse.

For monitoring of bacterial dissemination to the lungs and blood, mice were anesthetized. Blood was collected by cardiac puncture, and lungs were harvested and plated on BHIA supplemented with streptomycin (100 μg/mL, S6501, Sigma–Aldrich, Rehovot, Israel).

For the phage pharmacokinetic analysis, one dose of фA1122 or PST phage solution (1 × 10^9^ plaque-forming units (PFUs)/mouse) was IN administered to C57BL/6J mice (35 µL) or delivered by intraperitoneal (IP) injection (0.5 mL). Mice were anesthetized prior to IN administration. For phage enumeration in organs, mice (n = 3 per time point and per administration route) were sacrificed, and blood was collected by cardiac puncture followed by serial 10-fold dilution in SM buffer (0.1 M NaCl, 8 mM MgSO_4_, 50 mM Tris–HCl pH 7.5 and 0.01% gelatin solution). The lungs, spleen and liver were harvested, washed with PBS and transferred to 1 well in 6-well microplates. The spleen, liver and lungs were crushed in PBS (1 mL for the spleen and liver and 2 mL for the lungs). The lung extract was filtered through a 70 µm cell strainer. The crushed tissues were serially diluted 10-fold with SM buffer. Phage titration of the diluted samples was performed by the spot assay technique by dropping 3 drops of 10 µL samples on Kim53Δ70Δ10 lawns grown on BHIA top agar containing 200 µg/mL streptomycin to prevent bacterial contamination [25].

For bioluminescence imaging analysis of luciferase-expressing *Y. pestis*, mice were IN exposed to 10 × LD_50_ Kim53-*lux* followed by IN administration of фA1122 or PBS (control group). Photon emission from the lungs was visualized using an IVIS (Caliper Life Sciences, Hopkinton, MA, USA). Image acquisition was performed using the following settings: binning of 2 and acquisition times of 1–4 min. The luminescence signals for all images were normalized and reported as photons/second/cm^2^/sr using Living Image^®^ 4 software.

For titration of anti-F1 IgG in serum, blood was collected from mice via the tail vein at day 21 post infection (pi), and titers of IgG against F1 were determined using ELISA, as previously described [28].

### 2.3. Phage Treatment

Phages were applied as a single suspension (35 µL containing 10^9^ PFU of фA1122 or PST) or as a cocktail (фA1122 + PST, 10^9^ PFU each). IN administration was conducted at 5 h after *Y. pestis* infection. IP phage injections (0.5 mL, 1 × 10^9^ PFU/mouse) were conducted at 24 h intervals (regimens described in the relevant figure legend). Prior to IN phage administration, mice were anesthetized by subcutaneous injection of 0.5% ketamine HCl and 0.1% xylazine mixed solution. Control mice were administered PBS.

### 2.4. Antibiotic Treatment

Ceftriaxone (PHR-1382, Sigma–Aldrich, Israel) was injected subcutaneously twice per day for 5 consecutive days, starting at 48 h post *Y. pestis* infection.

### 2.5. Statistical Analysis

The results were analyzed by GraphPad Prism 8.2.0 software. Pairwise comparisons of mouse groups were performed using a log-rank (Mantel–Cox) test. A *p*-value of 0.05 was characterized as the significance threshold.

### 2.6. Bioluminescence-Based Lysis Assay

Blood from 3 naive C57BL/6J mice was pooled in citrate-containing tubes (Vacutainer sodium citrate tubes, BD, USA). The *Y. pestis* EV76-*lux* strain was grown on BHIA at 37 °C for 48 h. Bacterial colonies were suspended in PBS and inoculated (1:10; vol:vol) in BHI broth or in mouse whole blood and transferred (90 µL/well) into a 96-well transparent-bottom white microplate (Thermo Scientific Nunc: cat. no. 165306). Infection was performed by adding 10 µL of фA1122 or PST phage solution (MOI = 0.01) or 10 µL of SM buffer to the growth control wells. The bacterial growth curves were assessed by tracking the bioluminescent signal (relative light units, RLUs) of each well at 15 min intervals over 24 h using a SPARK 10M plate reader (Tecan, Männedorf, Switzerland). The temperature used in all experiments was 37 °C.

### 2.7. Phage Preparation and Purification

Purified bacteriophage stocks (фA1122 or PST phages) were prepared from phage-infected cultures of nonvirulent *Y. pestis* Kim53Δ70Δ10. Phage lysates were prepared by growing bacterial cultures in two 2 liter Erlenmeyer flasks containing 500 mL of BHI inoculated with an overnight starter culture to obtain an initial OD_600_ of 0.05. Bacterial cultures were incubated at 28 °C with shaking at 200 rpm until OD_660_ = 1. The phage solution was added to the bacterial culture (MOI = 0.01), and the culture was further incubated under the same conditions for another 4 h for фA1122 or 24 h for PST. Culture lysates were centrifuged at 6000 rpm for 10 min at 4 °C followed by 0.45 µm filtration for the removal of residual bacteria or bacterial debris. Bacterial DNA was removed by endonuclease digestion using DENARASE endonuclease (c-LEcta, Germany). Digestion was performed with 20 U/mL endonuclease in the presence of 2 mM MgCl_2_ (Spectrum, New Brunswick, NJ, USA) for 24 h at 4 °C under constant mild agitation. Sucrose (St. Louis, MO, USA) was added to the phage solution to a final concentration of 4%. Ultrafiltration was performed using a 300 or 750 kDa (for фA1122 or PST purification, respectively) nominal molecular weight cutoff (NMWC) with a 1 mm diameter polyethersulfone (PES) hollow fiber membrane cartridge (Cytiva, Marlborough, MA, USA). Using an ultrafiltration system, the phage solution was concentrated 10-fold by volume and diafiltrated X5 against PBS (Biological Industries, Beit Haemek, Israel) supplemented with 4% sucrose. The ultrafiltrated phage solution was filtered again through a 0.45 µm filter. Endotoxin removal was performed twice using a Toxin Eraser endotoxin removal kit (GenScript, Piscataway, NJ, USA) according to the manufacturer’s guidelines. Elution was performed with a PBS 0.4% sucrose solution buffer followed by 0.2 µm filtration. Residual endotoxins in the purified phage preparations were determined using the Limulus Amebocyte Lysate (LAL) Kinetic-QCL Kit (Lonza, Basel, Switzerland) according to the manufacturer’s instructions. Purified phage preparations containing endotoxin units (EU)/1E09 PFU ≤ 2 were used for the phage therapy experiments. The phage stock was stored at 4 °C in the dark until use.

## 3. Results

### 3.1. Intranasal Administration of фA1122 Leads to High Phage Titers in the Lungs

**Figure 1:**
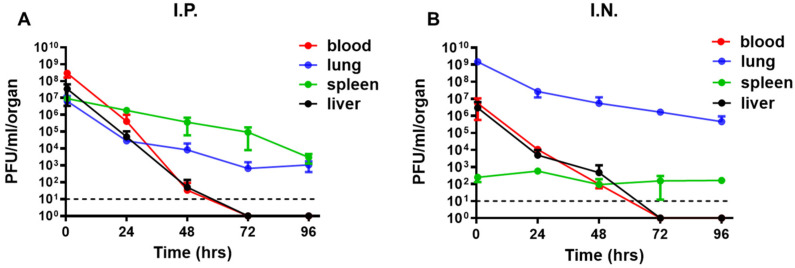
Pharmacokinetic analysis of фA1122 in naive mice. A single dose of фA1122 phage suspension (1 × 10^9^ PFU) was administered to naive C57BL/6J mice, either by (**A**) IP injection (0.5 mL) or (**B**) via the IN route (35 µL). For each administration route, n = 3 for each time point. Phage titration was performed using a spot assay test. Each dot represents the mean value in terms of PFU/organ (lung—blue dots, spleen—green dots, liver—black dots) or PFU/mL blood (red dots). Bars represent the standard deviations (SDs).

Pneumonic plague involves *Y. pestis* dissemination from the lungs to internal organs and blood [24]. Thus, for efficient phage treatment, phage distribution in those tissues is a prerequisite. Therefore, we tested the tissue distribution of the *Y. pestis*-specific lytic phage фA1122 in C57BL/6J mice and examined its dependency on the administration route. Mice were inoculated intranasally (IN) or intraperitoneally (IP) with a single dose of phage suspension (1 × 10^9^ plaque-forming units (PFU)/mouse). As shown in Figure 1, phages reached all tested tissues (blood, lung, spleen and liver) within 30 min. The IN route seemed advantageous for the treatment of pneumonic plague, as it led to the highest phage concentration in the lungs (−1 × 10^9^ PFU/lungs, Figure 1B). Moreover, high phage concentrations were maintained in the lungs for at least 4 days (Figure 1B). Thus, we further tested the potential of IN treatment with фA1122 against *Y. pestis* airway infection.

### 3.2. Intranasal Administration of a Single Dose of Phage Suspension Delayed Mortality in a Mouse Model of Pneumonic Plague

To evaluate the benefit of phage therapy, we IN administered a single dose of the фA1122 lytic phage (1 × 10^9^ PFU/mouse) at 5 h post IN infection with a lethal dose of 10 × LD_50_ *Y. pestis* strain Kim53.

**Figure 2:**
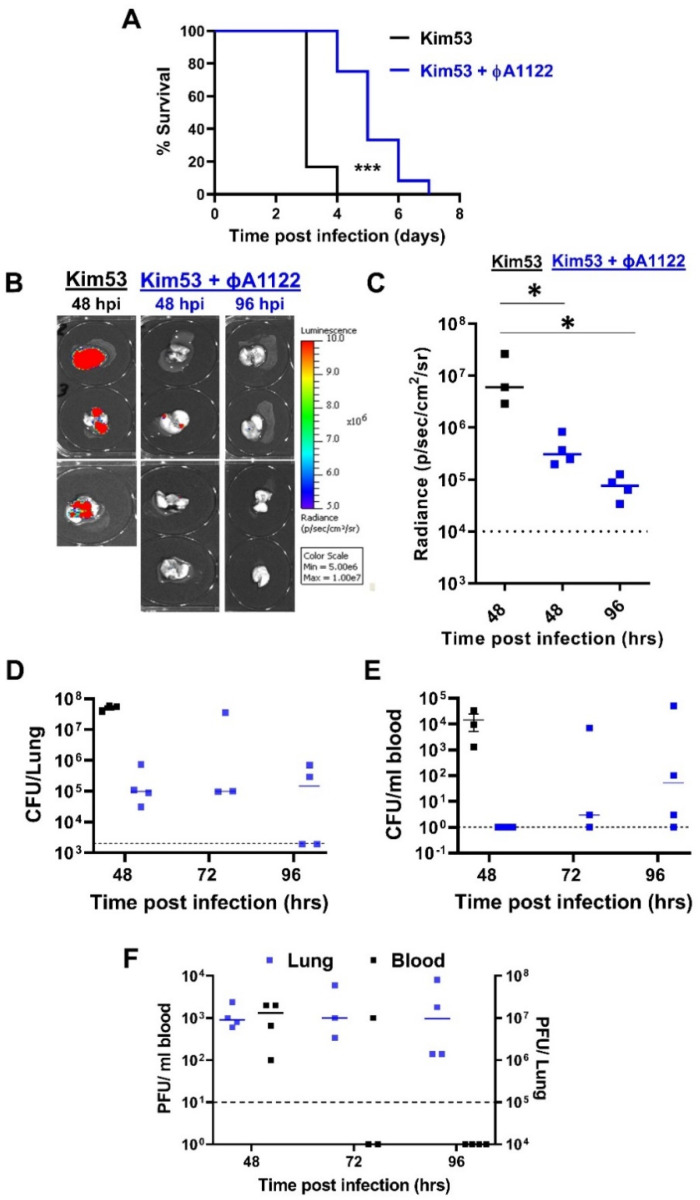
Intranasal administration of a single phage dose delayed disease progression. C57BL/6J mice were intranasally infected with 10 × LD_50_ *Y. pestis* Kim53 strain (**A**) or Kim53-lux luminescent strain (**B**–**F**). For phage treatment, one dose of 1 × 10^9^ фA1122 PFU/mouse was IN administered at 5 hpi. Control mice were subjected to intranasal administration of PBS. (A) Survival curves of control mice (black line, n = 6) and phage-treated mice (blue line, n = 12). (**B**,**C**) Control (n = 3) and phage-treated mice (n = 4) were anesthetized at the indicated time points post *Y. pestis* infection. Lungs were harvested, and imaging was performed using IVIS as detailed in the Section 2. (**D**,**E**) The bacterial load in the lungs and blood was quantified by plating serial dilutions of tissue homogenate/blood on BHIA plates supplemented with 200 μg/mL ampicillin and counting the colonies. Black squares represent nontreated control mice and blue squares represent phage-treated mice. (**F**) Phage titration was performed by the spot assay technique described in the Section 2. Each point represents the phage load in the lungs (blue squares, total PFU/lung) or blood (black squares, PFU/mL) of an individual mouse. Horizontal bars represent median values. Dotted lines mark the limit of detection. Statistically significant differences between groups are denoted by asterisks (* *p* < 0.05; *** *p* < 0.0001; log-rank (Mantel–Cox) test). Bars indicate standard errors of the means.

As shown in Figure 2A, *Y. pestis*-infected control mice died within 3–4 days post infection (pi), showing a mean time to death (MTTD) of 3 days. In contrast, phage-treated mice showed a significant delay in time to death, which occurred within 4–7 days pi, with a MTTD of 5 days.

To assess *Y. pestis* loads in the lungs of treated mice, we infected mice with a luminescent *Y. pestis* derivative (Kim53-*luxCDABE*), harvested the lungs at days 2 and 4 pi and visualized bacterial loads using an in vivo imaging system (IVIS). We found that at 48 h pi (hpi), lungs from the nontreated control mice showed significantly higher levels of luminescence than those from phage-treated mice (Figure 2B,C). Bacterial live counts measured at 48 hpi strengthened the IVIS results, revealing bacterial loads higher by more than two orders of magnitude in the control mice compared to the phage-treated mice (Figure 2D). Similarly, inhibition of *Y. pestis* growth in the lungs of phage-treated mice correlated with high concentrations of фA1122 (Figure 2D,F, respectively).

Next, we assessed *Y. pestis* dissemination in the blood. During the first 48 h, the bacterial concentration in the blood of the control mice reached −10^4^ colony-forming units (CFU)/mL, while no bacteria were detected in the blood of phage-treated mice (Figure 2E). However, in subsequent days, bacteria also appeared in the circulation of phage-treated mice (Figure 2E). The appearance of *Y. pestis* in the blood negatively correlated with the phage amount, which was low at 48 hpi and decreased further to under the limit of detection at 96 hpi (Figure 2F). We therefore concluded that while *Y. pestis* growth in the lungs of treated mice was restricted, the reduction in phage level in the blood during disease progression enabled *Y. pestis* dissemination/propagation in the circulation, which ultimately led to mouse death (Figure 2A).

### 3.3. Multiple-Dose Phage Administration Did Not Improve Treatment Efficacy

**Figure 3:**
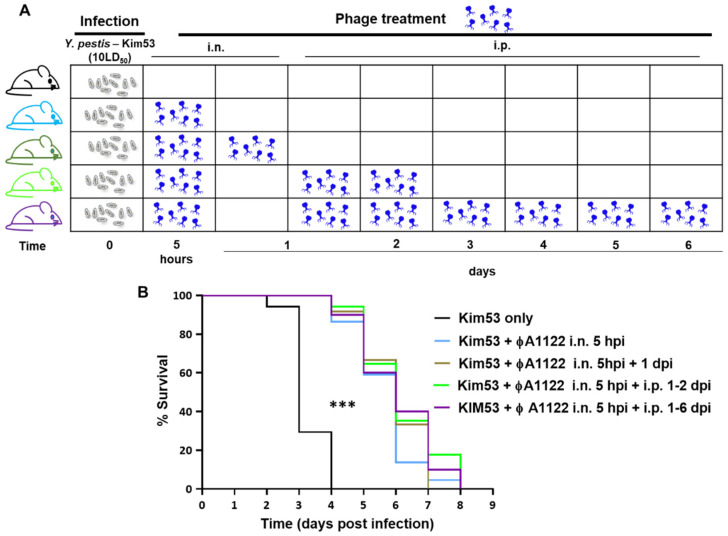
Multiple-dose фA1122 phage treatments did not improve treatment efficacy. Schematic representation of mouse treatment regimens (**A**) and survival curves (**B**). C57BL/6J mice were IN infected with 10 × LD_50_ of *Y. pestis* Kim53. For IN phage treatment, mice were inoculated with 35 µL of 1 × 10^9^ фA1122 PFU; IP injection included the administration of 0.5 mL of 1 × 10^9^ фA1122 PFU. The treatment regimens were as follows: one dose of IN фA1122 at 5 hpi (n = 22; light blue line), two doses of IN фA1122 at 5 hpi and 24 hpi (n = 12, olive green line), one dose of IN фA1122 at 5 hpi + IP injections at 24 and 48 hpi (n = 17, green line) and one dose of IN фA1122 at 5 hpi + IP injections for 6 days, every 24 h (n = 10, purple line). Control mice: n = 17, black line. (**B**): Statistically significant differences between the control group and phage-treated groups are denoted by asterisks (*** *p* < 0.0001; log-rank (Mantel–Cox) test).

To improve phage treatment and increase phage load in the blood and internal organs, we designed additional treatment regimens (Figure 3A). Pharmacokinetic analysis showed that in comparison to IN administration, IP injection of фA1122 led to higher phage concentrations in the blood, spleen and liver (Figure 1); thus, we added IP injection doses after the initial IN treatment. Moreover, since phages are rapidly cleared from the blood and liver (Figure 1), we provided serial phage treatment at 24 h intervals for 6 days pi. As depicted in Figure 3B, phage treatment was effective at delaying mouse mortality in comparison to the nontreated group, as shown above (MTTD = 6 and 3 days, respectively). However, no differences were found among the various phage-treated groups. Thus, increasing phage loads in internal organs during the course of the disease did not improve infection outcomes, suggesting that фA1122 lytic activity in the blood might be insufficient.

### 3.4. PST Phage Shows Improved Persistence and Activity in Mouse Blood Compared with фA1122

**Figure 4:**
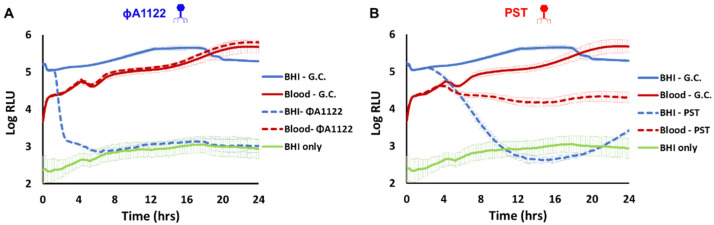
PST is more potent than фA1122 in the presence of blood. Phage-based lysis assays were performed with the bioluminescent *Y. pestis* strain EV76-lux (10^7^ CFU/mL) suspended in BHI broth (blue and orange lines) or in mouse whole blood (gray and red lines). The *Y. pestis* strain was infected with фA1122 (**A**) or PST (**B**) phages (10^6^ PFU/mL; multiplicity of infection (MOI) = 0.01). Bioluminescence (RLU) was measured at 37 °C in 15 min intervals for 24 h using a SPARK 10M plate reader. The experiment was performed in biological duplicates (using blood pooled from three mice for each experiment), and the results are representative of one experiment. Presented values are the average results from three wells in a single triplicate experiment, and the error bars represent the standard deviations (SD). The green line represents the background from BHI broth. G.C. = growth control.

Since the addition of IP administered phage doses did not improve mouse survival (Figure 3B), we tested the possibility that mouse blood may inhibit phage activity. We performed an in vitro lysis assay with фA1122 and an additional lytic phage, PST. As blood precludes the use of bacterial turbidity as a measure of lysis, we used a luciferase-expressing *Y. pestis* strain as the host and monitored the intensity of the bioluminescent signal in the presence or absence of фA1122 or PST. As shown in Figure 4, mouse blood inhibited фA1122 lytic activity, whereas the lytic activity of PST was preserved (although to a lesser extent than in BHI broth).

**Figure 5:**
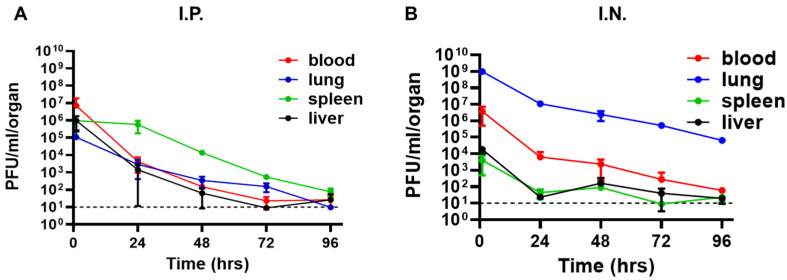
Pharmacokinetic analysis of PST in naive mice. A single dose of PST phage suspension (1 × 109 PFU) was administered to naive C57BL/6J mice, either by (**A**) IP injection (0.5 mL) or (**B**) via the IN route (35 µL). For each administration route, n = 3 for each time point. Phage titration was performed using a spot assay test. Each dot represents the mean value in terms of PFU/organ (lung—blue dots, spleen—green dots, liver—black dots) or PFU/mL blood (red dots). Bars represent the standard deviations (SDs).

As PST was advantageous over фA1122 in terms of its lytic activity in blood, we further evaluated its dissemination in mouse tissues following IN or IP administration. As shown in Figure 5, PST disseminated rapidly into mouse tissues following both IN and IP administration, as was observed for фA1122 (Figure 1). However, in contrast to the rapid clearance of фA1122 from the blood (to under the LOD by 72 h post administration; Figure 1), PST presence in the blood was detected even at 4 days post IN or IP administration. Notably, the spot assay was performed with blood samples diluted in buffer, therefore eliminating the inhibitory effect of blood on phage lytic activity and allowing the enumeration of infective phage particles.

### 3.5. Comparing the Protective Potential of PST and фA1122

**Figure 6:**
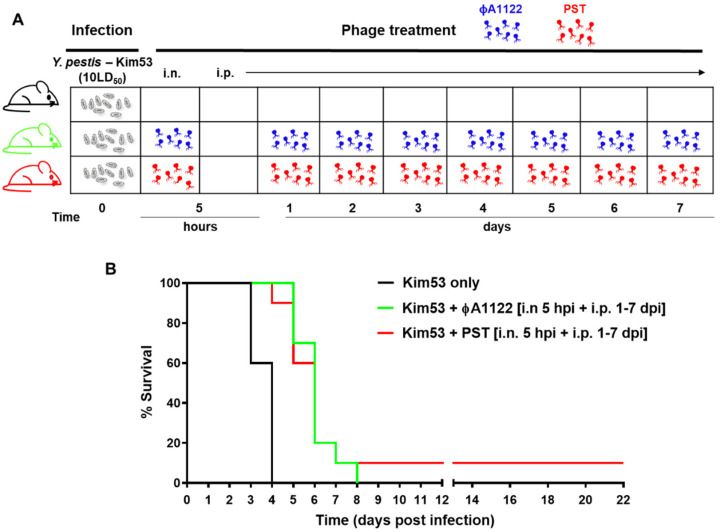
Treatment of pneumonic plague with фA1122 or PST results in similar outcomes. (**A**) Schematic presentation of the phage treatment regimen. C57BL/6J mice were IN infected with 10 × LD_50_ *Y. pestis* Kim53 (gray symbols), followed by PBS (control group) or phage administration. Each dose of PST (red phage symbol) or фA1122 (blue phage symbol) suspension contained 1 × 10^9^ phages. (**B**) Survival curves. The mouse groups were as follows: no phage (n = 5, black line), IN phage administration at 5 hpi followed by IP injections every 24 h on days 1–7 post bacterial infection (n = 10; green line for фA1122 and red line for PST).

Next, we compared the therapeutic potential of the phages. The treatment regimens with PST and фA1122 are schematically outlined in Figure 6A, and the results indicated that both phages had a comparable ability to delay the time to death (Figure 6B). This analysis indicates that phage therapy alone is limited in its ability to protect against acute pneumonic plague.

### 3.6. Phage–Ceftriaxone Combination Therapy Is Highly Effective against Pneumonic Plague

**Figure 7:**
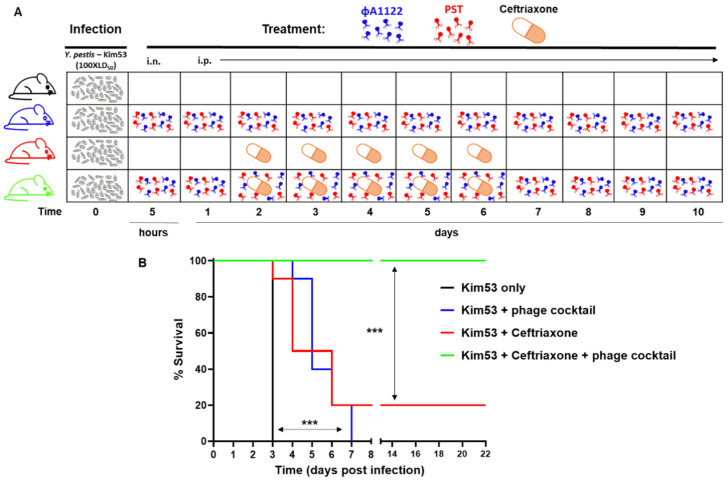
Effective rescue of infected mice by the phage–antibiotic combination treatment. Schematic presentation of the treatment regimens (**A**) and survival curves (**B**) Female C57BL/6J mice were IN infected with 100 × LD_50_ *Y. pestis* Kim53. Mouse groups included control nontreated (n = 4), phage-treated (n = 10), ceftriaxone-treated (n = 10) and phage–ceftriaxone combination-treated mice (n = 9). Phage treatment was performed using a phage cocktail composed of фA1122 and PST (1 × 10^9^ each phage/dose; 35 µL for intranasal administration or 0.5 mL for IP injection). Treatment included IN administration at 5 hpi followed by IP injections at days 1–10, with 24 h intervals. Ceftriaxone was subcutaneously injected every 12 h on days 2–6 post bacterial infection. Mice were monitored for 22 days. Statistically significant differences are denoted by asterisks (*** *p* < 0.001; log rank (Mantel–Cox) test).

In the event of a plague outbreak involving MDR bacteria, second-line antibiotics may be used as an alternative treatment. Cephalosporins are broad-range antibiotics that are active against *Y. pestis* strains in vitro [1], but they are limited in their activity in vivo [29,30]. To assess the therapeutic value of adjunctive phage therapy to second-line antibiotic treatment, C57BL/6 mice were IN challenged with a high dose of *Y. pestis* Kim53 (100 × LD_50_) and treated with phage, ceftriaxone or a combination of both (Figure 7A). For phage treatment, we used a cocktail composed of both фA1122 and PST (1 × 10^9^ PFU of each phage per dose). Antibiotic treatment with ceftriaxone rescued only 20% of the mice (Figure 7B), and similar to treatment with PST or фA1122 alone (Figure 6), treatment with the phage cocktail delayed mortality; however, all mice succumbed to the infection (Figure 7B). In contrast, the phage–antibiotic combination treatment was highly effective, leading to the survival of all infected animals (Figure 7B) and clearance of the pathogen from the spleens of all surviving animals at day 21 pi. In addition, surviving animals developed high serum IgG antibody titers against the protective F1 antigen (GMT = 25,000 at 21 days pi), implying that the combined treatment also enabled the development of adaptive immunity toward *Y. pestis*.

## 4. Discussion

The experience of the current emerging COVID-19 pandemic with its broad effects on humankind has highlighted the need for preparedness for pandemics. In this regard, a major concern is an outbreak caused by highly transmissible MDR pathogens for which current antibiotics may become ineffective. In the case of the emergence and spread of natively derived or deliberately engineered plague-causing *Y. pestis* MDR strains, clinicians will have to use less effective second-line antibiotics for treatment. Adjunct phage therapy may improve the efficiency of such second-line antibiotics and serve as a safe alternative treatment in the event of MDR infections.

The potential of phages as an anti-plague therapy was reported almost a century ago (in 1925) by Felix D’Herelle, who injected *Y. pestis*-specific phage preparations into the buboes of four patients with plague, all of whom recovered [31]. However, following inconclusive results obtained with additional phage treatment experiments and with the availability of effective antibiotics, interest in developing phage-based therapy against plague decreased. In one of the few studies in which the effectiveness of phage therapy against plague was examined, Filippov and his colleagues assessed the outcome of phage therapy with the фA1122 phage in a mouse model of bubonic plague. The study demonstrated that phage treatment was able to delay mouse mortality and provide protection to some of the infected animals [32].

In the present study, we explored the potential of phage therapy (фA1122 and PST) alone and in combination with the second-line antibiotic ceftriaxone against pneumonic plague using a mouse model. The *Y. pestis*-specific lytic phages фA1122 and PST were chosen for treatment as they are well characterized and their genomic sequence is known and does not contain genes encoding integrase, known antibiotic resistance proteins or toxins [21,22]. The фA1122 phage is well suited for use as treatment, as it was shown to be able to infect thousands of *Y. pestis* strains and thus is used by the CDC and the U.S. Army Medical Research Institute of Infectious Diseases for *Y. pestis* diagnostics [22,33]. In addition, фA1122 and PST bind to different receptors, the Kdo/Ko moiety in the LPS molecule for фA1122 and the Hep(II)/Hep(III) of LPS for PST, making their combination a preferable cocktail to prevent the selection of phage-resistant mutants [21,22,26]. Moreover, mutations in the *Y. pestis* LPS phage receptors attenuate *Y. pestis* virulence [21,22]; therefore, the development of phage resistance is less likely.

We have shown here that treatment with phage only delayed MTTD and did not rescue *Y. pestis*-infected mice (Figure 2, Figure 3 and Figure 6). As the enrichment of circulating phages did not improve the survival rate, this may indicate a reduction in phage lytic activity in the presence of mouse blood, as we indeed observed in the in vitro lysis assay (Figure 4). This reduction in phage activity in the presence of blood may be explained by several mechanisms, including interference with the phage binding to its specific bacterial receptor by blood components. Another possibility is that alterations of membrane and cellular protein composition may affect phage propagation and lytic activity. We also recently observed a reduction in фA1122 lytic activity toward *Y. pestis* suspended in human blood [25] and this finding has been reported for other phages [34,35]. However, the PST phage, which retained in vitro activity in the presence of blood, still yielded a similarly low therapeutic efficiency in vivo, as observed for фA1122 in vivo (Figure 6), indicating that additional limiting factors exist.

The presence of фA1122 and PST in the spleen and the liver, as reflected by the pharmacokinetic analysis, may indicate phage clearance by the reticuloendothelial system, whereby phages are not free to infect bacteria [36]. Similar observations for фA1122-treated mice with bubonic plague led Filippov and his colleagues to suggest that the phage and pathogen reside in different compartments in the spleen and liver; therefore, the phage is unable to infect and clear the bacterium [32,37].

Finally, as we show here, фA1122 and PST are differentially inhibited by (unknown) blood/internal organ-derived component/s. These differences may result from the difference in their targeted receptors on the bacterial surface; some components may interfere with some receptors and not with others. Improving phage binding to its host bacteria in the presence of blood or internal organ extracts may improve phage therapy outcomes, especially in the case of systemic bacterial diseases. Modulating the phage tail tip by either genetic engineering [38] or by a continuous evolution system [39] may alter the phage receptor and enhance its binding to its host bacteria.

Although phage treatment failed to save the infected animals, the significantly delayed mortality encouraged us to evaluate the potential of phage as an adjunct therapy to second-line antibiotics as an alternative countermeasure against MDR *Y. pestis*. Various case reports and animal model studies have shown that combining antibiotics with phages may enhance bacterial clearance, increase antibiotic penetration into biofilms and decrease the emergence of phage resistance (see reviews [40,41]).

For the combined phage–antibiotic treatment, we used a phage cocktail composed of фA1122 and PST, gaining the high observed efficiency of фA1122 to eliminate *Y. pestis* in the lungs and PST lytic activity in the presence of blood. Additionally, фA1122 and PST bind to different sites on the LPS molecule [21,26] and thus target different receptors. Phage cocktails composed of several lytic phages targeting various receptors are recommended for efficient phage therapy, as this approach broadens the host range and decreases the probability of phage resistance evolution [36,42].

The antibiotic used in the combined treatment was ceftriaxone, a cephalosporine class of antibiotics that inhibits cell wall synthesis. Ceftriaxone has been shown to display potent in vitro activity against *Y. pestis* strains [43] but limited ability to provide protection in a mouse model of pneumonic plague [29,30]. Indeed, when mice were treated with ceftriaxone as a single treatment, 80% of the animals succumbed to the infection (Figure 7B). In contrast, the combined treatment of ceftriaxone and the phage cocktail was highly effective, leading to the survival of all infected animals (Figure 7B) and clearance of the pathogen from internal organs. Surviving animals developed high antibody titers against the protective F1 capsular antigen, indicating the development of adaptive immunity that probably contributed to the combined treatment efficacy.

In conclusion, our results suggest a synergistic effect between phage and antibiotic treatment and highlight the potential of these combinations for emergency use even in cases of acute infections, such as pneumonic plague. Notably, training phages to efficiently lyse bacteria in the presence of blood components may improve therapeutic outcomes.

Further research on phage combinations with antibiotics and the potential for resensitization to antibiotics may broaden the efficacy of phage therapy as an alternative therapy for MDR pathogens.

## Figures and Tables

**Table 1:** *Y. pestis* strains used in the present study.

Strain	Use	Virulence	Reference
Kimberley53 (Kim53)	Challenge in animal experiments	+	[23]
Kimberley53-*luxCDABE* (Kim53-*lux*)	Imaging of bacteria in the lungs (by in vivo imaging system/ IVIS)	+	[24]
Kim53ΔpPCD1ΔpPCP1 (Kim53Δ70Δ10)	Phage preparation and titration	-	[25]
EV76-*luxCDABE* (EV76-*lux*)	Phage lysis assay	-	[25]

## Data Availability

Not applicable.
